# The development of a healing model of care for an Indigenous drug and alcohol residential rehabilitation service: a community-based participatory research approach

**DOI:** 10.1186/s40352-017-0056-z

**Published:** 2017-12-04

**Authors:** Alice Munro, Anthony Shakeshaft, Anton Clifford

**Affiliations:** 1 National Drug and Alcohol Research Centre, University of New South Wales, Sydney, NSW 2052 Australia; 2 University of Queensland, Brisbane, QLD 4072 Australia

**Keywords:** Indigenous drug and alcohol residential rehabilitation, Criminal justice system, Community-Based Participatory Research, Remote, Model of care, Research partnerships

## Abstract

**Background:**

Given the well-established evidence of disproportionately high rates of substance-related morbidity and mortality after release from incarceration for Indigenous Australians, access to comprehensive, effective and culturally safe residential rehabilitation treatment will likely assist in reducing recidivism to both prison and substance dependence for this population. In the absence of methodologically rigorous evidence, the delivery of Indigenous drug and alcohol residential rehabilitation services vary widely, and divergent views exist regarding the appropriateness and efficacy of different potential treatment components. One way to increase the methodological quality of evaluations of Indigenous residential rehabilitation services is to develop partnerships with researchers to better align models of care with the client’s, and the community’s, needs. An emerging research paradigm to guide the development of high quality evidence through a number of sequential steps that equitably involves services, stakeholders and researchers is community-based participatory research (CBPR). The purpose of this study is to articulate an Indigenous drug and alcohol residential rehabilitation service model of care, developed in collaboration between clients, service providers and researchers using a CBPR approach.

**Methods/Design:**

This research adopted a mixed methods CBPR approach to triangulate collected data to inform the development of a model of care for a remote Indigenous drug and alcohol residential rehabilitation service.

**Results:**

Four iterative CBPR steps of research activity were recorded during the 3-year research partnership. As a direct outcome of the CBPR framework, the service and researchers co-designed a Healing Model of Care that comprises six core treatment components, three core organisational components and is articulated in two program logics. The program logics were designed to specifically align each component and outcome with the mechanism of change for the client or organisation to improve data collection and program evaluation.

**Conclusion:**

The description of the CBPR process and the Healing Model of Care provides one possible solution about how to provide better care for the large and growing population of Indigenous people with substance.

## Background

The aetiology of the harmful effects of substance misuse on Indigenous Australians is a complex range of factors including the intergenerational impacts of colonisation and subsequent high rates of incarceration, suicide, self-harm and poverty (Wynne-Jones et al., 2016; Marmot, 2011; Productivity Commission, 2016; DoHA, 2013; ACOSS, 2016). Indigenous Australians comprise approximately 3% of the Australian population (ABS, 2014), and drug and alcohol-related morbidity and mortality are disproportionately higher among this population (AIHW, 2011; AIHW, 2016). In order to further reduce rates of substance misuse harms, more effective prevention and treatment programs that are tailored to the specific needs of Indigenous Australians are required.

Indigenous drug and alcohol residential rehabilitation services are a preferred option for Indigenous people who have high levels of substance dependence, primarily because they provide a culturally acceptable form of treatment (Brady, 1995; Chenhall & Senior, 2013). In addition to being culturally acceptable, Indigenous residential rehabilitation services are typically multi-component, reflecting the complex social, economic, housing, mental health, crime and legal challenges experienced by their clients (Wilson et al., 2017; Honorato et al., 2016; Leal et al., 1998; Farabee & Shen, 2004; Brunette et al., 2004; Mortlock et al., 2011; Weatherburn, 2008). A current analysis of the characteristics of clients admitted to a remote Indigenous residential rehabilitation service in NSW, Australia, for example, highlighted the strong correlation between their significant health and socio-economic needs, and their involvement in the criminal justice system (Munro et al., 2017, under review). This analysis not only showed that the majority of clients were referred from the criminal justice system, but that this proportion had statistically significantly increased over time, from 79% in 2011/12 to 96% in 2015/16. Most clients had at least two co-occurring risk factors, in addition to a criminal history: 69% self-reported polysubstance use (primarily methamphetamines, alcohol and cannabis) and 51% reported a current mental illness (primarily depression, anxiety and bipolar disorder). The statistically significant growth in clients referred from the criminal justice system is consistent with the reported 77% increase in adult Indigenous prisoners in Australia from 2000 to 2015 (Productivity Commission, 2016) and the disproportionately high prevalence of substance misuse among prisoners, which has been identified as a key driver in the disproportionately high incarceration rate (Weatherburn, 2014; Indig et al., 2010; Doyle et al., 2015; NIDAC, 2014; Weatherburn, 2008).

Given the well-established evidence of disproportionately high rates of substance-related morbidity and mortality after release from incarceration (Kinner et al., 2011), access to comprehensive, effective and culturally appropriate residential rehabilitation treatment will most likely assist in reducing recidivism to both prison and substance abuse for Indigenous Australians (NIDAC, 2014; Kinner & Wang, 2014; Heffernan et al., 2016). The 2015–16 Aboriginal and Torres Strait Islander Online Services Report (OSR) from Australia, however, identified a number of gaps in current service provision, particularly in relation to addressing the mental health and the social and emotional wellbeing needs of Indigenous clients (AIHW, 2017). Further, despite the need to establish the relative effectiveness of different configurations of culturally acceptable, multi-component treatments delivered in Indigenous residential rehabilitation services, a current systematic review of studies of Indigenous residential rehabilitation services from Australia, the United States, Canada and New Zealand, published between 2000 and 2016, identified only one quantitative evaluation (James et al., 2017, under review). This finding is consistent with results from a recent bibliometric review of published literature from the Indigenous drug and alcohol field generally, which found evaluations represented only 11% of published research in the past twenty years for Australia, the United States, Canada and New Zealand (Clifford & Shakeshaft, 2017). These reviews emphasise the need for more rigorous evaluations of Indigenous drug and alcohol services, including residential rehabilitation treatment.

In the absence of sufficient evidence from quantitative evaluation studies about the most cost-effective configurations of multi-component treatments, approaches to the delivery of Indigenous residential treatment programs vary widely, and divergent views exist regarding the effectiveness and appropriateness of different potential treatment components. As such, specific, evidence-based features of Indigenous residential programs are not well defined (James et al., 2017, under review; Chenhall & Senior, 2012; Chenhall & Senior, 2013; Gone & Calf, 2011; Taylor et al., 2010). One way to increase the quantity and methodological quality of evaluations of Indigenous residential rehabilitation services is to develop collaborative partnerships between services and researchers, to work together to develop models of care that synthesise the views of clients and service providers with existing research evidence, including both descriptive data and evaluations of treatment outcomes (Shakeshaft et al., 2012). Identified as a key priority in the 2014–19 National Aboriginal and Torres Strait Islander Peoples Drug Strategy (NDS, 2015), such partnerships could simultaneously co-create new knowledge and optimise client outcomes by embedding the development and evaluation of treatment models into the routine delivery of services. The purpose of this study is to report on the articulation of a model of care for an Indigenous drug and alcohol residential rehabilitation service, developed in collaboration between clients, service providers and researchers.

## Methods

### Ethics approval and consent to participate

Ethical approval was sought and granted by the Aboriginal Health and Medical Research Council (1023/14) and the University of New South Wales Human Research Ethics Committees (HC14142).

### Setting and clients

This study was undertaken with Orana Haven Aboriginal drug and alcohol residential rehabilitation service (OH), which is located in NSW, approximately 700 km north-west of Sydney (in relation to OH, the word Aboriginal is used because it is recommended by the Aboriginal Health and Medical Research Council as being most appropriate for the Indigenous peoples of NSW). The service began operating as an Aboriginal Community Controlled Health Organisation (ACCHO) in 1983. OH’s current vision builds on this long history of Aboriginal community-control, and that is to “provide a culturally safe drug and alcohol healing centre that maximises the strengths of Aboriginal people and communities” (OH 2015–2018 Strategic Intent, Supp. 1). Based on a combination of a Therapeutic Community and 12-Step treatment approach, OH offers a 3-month voluntary rehabilitation program for Aboriginal males, 96% of whom were referred from the criminal justice system in 2015/16. OH has an average of 66 client admissions annually, of whom 85% identify as Aboriginal. Mean length of stay is 56 days, although a third (36%) discharge within the first month. An estimated 32% of clients complete the program, 47% self-discharge and 20% are house-discharged for failing to comply with treatment requirements, such as providing continuously clean urine samples. OH’s completion rate of 32% is comparable to the 34% reported for non-Aboriginal residential rehabilitation services in Australia (Darke, Campbell & Popple, 2012), but it is possible this could be improved given the 62% completion rate reported in another study (Sung, Belenko & Feng, 2001). Due to inconsistent reporting across Indigenous residential rehabilitation services, rates of self-discharge could not be reliably compared with OH’s average of 47% of all clients.

### Study design

**Fig. 1:**
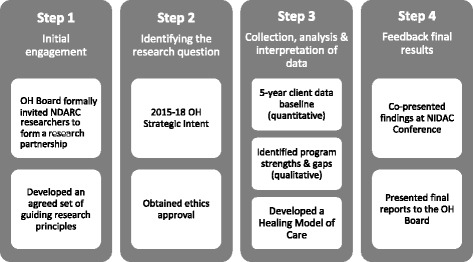
The community-based participatory research (CBPR) approach for Orana Haven

This 3-year (2014–2017) study used a community-based participatory research (CBPR) approach. CBPR is an emerging transformative research paradigm designed to bridge the gap between science and practice through community or service provider engagement throughout the research process, to achieve social change (Lazarus et al., 2014; Windsor, 2013; Wallerstein & Duran, 2006). The process of CBPR typically involves cycles of collaborative action, often in sequential steps that engage community or service provider participants as co-researchers, educating and empowering them to effect positive changes in their environment (Kowanko et al., 2009; Windsor, 2013; Lazarus et al., 2014). Given CBPR does not outline a specific and rigorous methodology, however, Windsor (2013) proposes the addition of mixed scientific methods to ensure adequate rigor in the production of new knowledge. In the context of Indigenous health, CBPR has been shown to be highly culturally acceptable (Mooney-Somers & Maher, 2008; Cochran et al., 2008; Pyett, 2002; Snijder et al., 2015). As visually represented in Fig. 1, the CBPR framework designed for this study comprised four iterative steps.

#### Step 1: Effective engagement (March 2014 - October 2014)

The activities that facilitated effective engagement were:

i) *A formal invitation from OH’s Board of Directors to the National Drug and Alcohol Research Centre (NDARC) to form a partnership.* In 2014, OH received federal funding to evaluate their treatment program and undertake capital works. The funding provided scope for OH to independently engage with experts and, consequently, OH’s Board of Directors invited the National Drug and Alcohol Research Centre (NDARC) to partner with them to review their treatment program.
ii) *An initial meeting between the OH Board and NDARC researchers to define the scope of the proposed evaluation and the principles of the partnership.* It was agreed that this meeting should be face-to-face, held on OH’s premises (to accommodate the clinical and administrative processes of OH and provide an opportunity for researchers to tour the service), and involve senior academics (professorial level) and junior researchers to reflect the seniority of OH’s Board membership.
iii) *The joint development of a set of guiding principles for the partnership.* These principles were further developed and agreed subsequent to the initial meeting, and were designed to be consistent with the National Health and Medical Research guidelines (NH&MRC, 2003) and the Australian Institute of Aboriginal and Torres Strait Islander Studies Guidelines for Ethical Research in Indigenous Studies (AIATSIS, 2012):
  1. Mutual recognition that meaningful change takes time. Consistent with the CBPR approach with Indigenous communities, both NDARC and OH allowed considerable project lead-time to understand the strengths and expertise from both sides of the partnership and build mutual trust.
  2. Regular, scheduled meetings. Both partners agreed that good communication is the foundation for a successful, long-term research partnership. As such, an agreed schedule of visits to the OH service, meetings with the Board of Directors and regular teleconferences with key stakeholders and community leaders was implemented so that researchers and OH stakeholders had open dialogue about the research process.
  3. The research activity should be closely tied to OH’s strategic planning needs and make a significant contribution to new knowledge. This principle ensured that the research was beneficial for both OH and the researchers.
  4. Sharing ownership over the project. In recognition of OH’s co-leadership of the research process, site-visits were specifically organised to be flexible and responsive to the demands of the service and Board members (especially when unexpected cultural obligations occurred), rather than only the schedules of the researchers. It was also accepted by the OH Board that formal research requirements (such as ethics approvals) were lengthy processes and needed realistic timeframes.

#### Step 2: Identifying the scope of the research (November 2014 – September 2015)

The activities that determined the specific nature of the research questions were:

i) *NDARC researchers agreed to assist OH develop its strategic intent for 2015-2018.* OH invited the researchers to assist them in developing their strategy to meet the National Safety and Quality in Health Care (NSQHC) Standards, which was closely aligned with the revision of OH’s strategic plan. The researchers considered this was a unique opportunity to: i) better understand the service’s specific needs; ii) deepen the process of engagement and trust, as outlined in Step 1; and iii) apply robust research methods to create rigorous new knowledge that would both inform OH’s strategic plans and engender publications for the peer-reviewed, academic literature. The strategic planning process involved conducting two focus groups, between May-July 2015, with OH staff and the Board of Directors. Data from the focus groups were analysed using thematic analysis, which identified three strategic priorities: 1) strong governance and sustainability; 2) supported and skilled staff; and 3) effective, culturally safe service delivery. The 2015-2018 Strategic Intent was presented to the Board for feedback and subsequent approval in September 2015, and supported OH’s successful NSQHC accreditation in November 2015.
ii) *Generating a clear research protocol for ethics approval.* Researchers and OH staff worked collaboratively to co-design the detailed mixed-methods research protocol. The purpose of this protocol was to obtain clarity and agreement about the required research methods for approval by the OH Board, the local ACCHOs and the appropriate research ethics committees. This process required 12 months to complete.

#### Step 3: Collection, analysis and interpretation of the data (October 2015 – October 2016)

##### Quantitative data

Researchers worked in partnership with OH staff to collect, analyse and interpret client and service data collected at OH during a 5-year period from 1 May 2011 to 30 April 2016. Two processes for collecting quantitative data were implemented at OH. First, client details were hand-written into a service admission book upon intake and discharge. Data collected included: demographics; referral type; and service utilization characteristics (e.g. type of discharge, length of time in treatment). Second, after a recommendation from researchers to obtain additional client information to inform service delivery, OH staff took the initiative to develop and implement a phone assessment form from 2015 to 2016 to better understand the health, psychological and social status of clients admitted to the service. Data collected included: previous rehabilitation service experience; previous and current legal history; drug and alcohol history; current income; and current physical (e.g. asthma, diabetes) and mental health diagnoses (e.g. bipolar disorder, depression). As this self-report phone assessment was a service-designed tool, no validation of this measure has been undertaken. A combination of this baseline data was analysed to better understand client characteristics and improve local decision-making to better tailor the service to client needs and has been published elsewhere (Munro et al. 2017, under review). Preliminary results were fedback to OH staff at two separate Board meetings (in February and August 2016) to facilitate collaborative interpretation of the data to ensure outcomes were clinically meaningful.

##### Qualitative data

Researchers adopted purposive sampling (Barbour, 2001) to conduct a total of 21 in-depth, semi-structured interviews with OH nine staff and twelve clients. The semi-structured interviews used ‘yarning’ approach, a form of culturally respectful conversation that is relaxed, narrative-based and emphasises the value of storytelling (Bessarab & Ngandu, 2010). Interviews were conducted across two phases (<3 months apart) to ensure qualitative data was captured at different time intervals. Interviews were conducted by a female non-Aboriginal researcher (AM) at OH, were digitally recorded, and later transcribed by an external transcriber to minimise researcher bias. Interview data were analysed using Interpretative Phenomenological Analysis (IPA) methodology, the findings of which are published elsewhere (Munro et al., 2017, under review).

#### Step 4: Feedback of final results (November 2016 – June 2017)

A dissemination process of the final results from the current CBPR study occurred in two ways. First, the primary author and a senior Aboriginal drug and alcohol worker from OH had the opportunity to co-present findings at the 2016 National Indigenous Drug and Alcohol Conference (NIDAC), the most notable Indigenous drug and alcohol conference in Australia. The value of OH as a culturally safe and effective treatment service in remote Australia was recognised by OH being presented with the NIDAC Service Recognition Award. In addition, a senior OH staff member was also recognised for their years of service at OH with the NIDAC Remote Male Worker Award. Second, final reports were presented for feedback and subsequent approval at two separate OH Board meetings in April 2017 and June 2017, thus completing Step 4 of the CBPR process.

## Results

A triangulation of the following sources of data informed the Healing Model of Care described in the results: i) Focus groups; ii) Quantitative data; and iii) Qualitative data. First, the focus groups identified key strategic priorities for OH in addition to the need for strong and transparent governance. Second, the quantitative data identified the most prevalent client characteristics, to which the Healing Model of Care ought to be tailored: clients were mostly Aboriginal men, all had multiple risk factors, were mostly referred from the criminal justice system, and were mostly aged from 26 to 35. Third, the qualitative data identified the importance of a structured program, the value of therapeutic relationships and the critical importance of healing by immersion in Aboriginal culture and being on traditional “country.” The term “country” is often used by Australian Indigenous people to describe the complex and interrelated connections to family origins in Australia and the Torres Strait (QSA, 2008). This includes the geographical region where a person’s family is from and their connections to this region and its people.

### Healing Model of Care

**Fig. 2:**
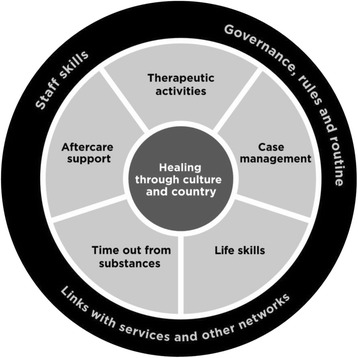
Core components of Orana Haven

The Healing Model of Care is comprised of the following:

1. Core components of OH, as summarised in Fig. 2 and detailed in the text below; and
2. OH treatment and organisational program logics, as summarised in Tables 1 and 2.

**Table 1:** Orana Haven Treatment Project Logic

a. Client areas of need	b. Intervention		c. Mechanisms of change	d. Process measures	e. Outcomes*
	Core treatment components	Flexible activities			
Primary client areas of need:	Healing through culture and country	- Being on country/spiritualty	Reconnecting clients to culture and country via activities and strong relationships	No. of clients engaged in regular cultural activities	Primary outcomes:
1. Risky substance use					1. Reduced substance misuse (AUDIT* / IRIS* clean urines*)
		- Developing kinships			
		- Making artefacts, fishing bush medicine			
2. Poor quality of life	Case management	- Referrals to local health services and visiting specialists	Clients engaged in the program via positive therapeutic alliance between staff and clients	No. of clients staying in the program for 3 or more mths	2. Increased quality of life (WHOQoL-BREF*)
3. Poor cultural connection					
					3. Increased connection to culture (GEM*)
		- Working with corrections	Referrals to AMS to external health and other social services	No. of Indigenous Health Checks/other referrals	
		- File notes / assessments			
		- Client transport		No. of kms of transport	
Secondary client areas of need:	Therapeutic activities	- One-on-one counselling	Improving client quality of life	No. of clients maintaining abstinence 3 months post discharge	Secondary outcomes
4. Co-occurring mental illness		- AA, morning, psychoeducational groups	Increased understanding of substance misuse (e.g. triggers) and personal strategies (e.g. motivations, goals, timeout) for reducing misuse		4. Reduced psychological distress (IRIS* / K10*)
5. Criminal justice involvement					
		- Informal counselling		No. of external counselling sessions provided	5. Reduction in recidivism (Pre/post criminal justice data)
6. Chronic physical health needs	Life skills	- Develop daily routine	Reconnecting clients to culture and country	No. of vocational-related courses completed	6. Improved physical health (Pre/post Indigenous health check outcomes)
7. Tobacco use		- Positive role-modelling	Relearning daily routine and structure to maintain a healthy lifestyle after discharge	No. of clients achieving individualised life skills goals	
					7. Reduction in smoking (RBD Scale* / self-report* / CO levels*)
8. Unemployed / limited education					
		- Redevelop personal responsibility			8. Improvement in employment and education (3mth follow-up data)
		- Vocational courses	Learning and developing work-ready and communication skills		
		- Literary/ communication skills			
	Time out from substances	- Improve physical wellbeing (eg. sleep routine / nutrition)	Identify and engage in positive alternative activities to substance use to learn how to take time out from substance substances	No. of clients engaging in regular exercise / cultural activities	
		- Improve physical wellbeing (eg. sleep routine / nutrition)			
				No. of clients quitting or reducing smoking	
		- Smoking cessation			
		- Referrals to services post-discharge (eg. ACCHOs)	Continue to access treatment and care required to maintain improved health and wellbeing post discharge	No. of clients maintaining abstinence/not involved in crime post discharge	
		- Provide a list of support services in client’s community (eg. AA)	Developing aftercare program post discharge from treatment		
		- Ongoing phone contact			

*Measured at admission, mid, discharge and 3mths post discharge from the OH program

**Table 2:** Orana Haven Organisational Program Logic

a. Organisational areas of need^a^	b. Treatment		c. Mechanisms of change	d. Process measures	e. Outcomes
	Core organisational components	Flexible activities			
1. Effective culturally safe service delivery	Links with services and other networks	- Partnerships with local services	Ongoing strong partnerships with local service providers and external networks	Type and no. of services or programs integrated into OH service delivery	Improved primary and secondary client outcomes (Table 1)
		- Networks across the field (eg. NADA, Bila Muuji)			
			Regular CQI feedback to inform local decision making	No. of network meetings attended	
		- CQI cycles and capacity building			
2. Supported and skilled staff	Staff skills	- Staff must be client-centred	Client-centred staff committed to improving client outcomes	No. of staff training completed	Improved client intake/discharge data
		- Regular staff training	Pathways to increase and up skill Aboriginal staff at OH	No. of Aboriginal staff employed at OH	Improved staff retention
		- Regular clinical and cultural supervision			
			Staff are supported by OH via regular clinical and cultural supervision and access to training	No. of staff receiving cultural/clinical supervision	
3. Strong governance and sustainability	Governance, rules and routine	- Regular Board meetings	Strong vision and purpose of OH program	No. of Board meetings	Program Accreditation
				No. of staff meetings	
		- Annual review strategic intent to meet ongoing accreditation standards	Local decision making from an empowered Board and community		Current OH Strategic Intent
				Annual budget	
				Annual review of treatment and organisational process measures	Annual reports to stakeholders and funders
			Regular governance training and inductions for Board members		
		- Consistent program rules/routine for clients and staff			
					Ongoing economic analysis (eg. Cost Benefit Analysis)
				No. of capital works/maintenance projects	
		- Strong regional advocacy			
			Capital works/maintenance projects		
		- Ensure adequate resources and ongoing capital works			
				No. of kms of transport	
			Ongoing partnerships with researchers and funding bodies to ensure adequate resources		
		- Regular feedback of program outcomes to staff, Board, community/stakeholders via reporting systems			

^a^Organisational areas of need obtained from three strategic priorities specified in the 2015–18 OH Strategic Intent

#### Core components of OH

Figure 2 delineates two broad areas of OH’s service delivery. First, the two centre circles represent the six core treatment components. Second, the black outer circle represents the core three organisational components. The central component of OH’s treatment service delivery is *healing through culture and country*, which is why it is shown in the centre of Fig. 2. The other five core treatment components enable healing through culture and country, shown in the middle section of Fig. 2, and includes*: therapeutic activities; case management; life skills; time out from substances;* and *aftercare support.* The effective delivery of these treatment components is dependent upon the three core organisational components, as shown in the outer circle of Fig. 2: *governance, rules and routine; staff skills;* and *links with services and networks*. A detailed description of these components is provided below.

##### Healing through culture and country

There are a number of activities that operationalise the centrality of *healing through culture and country*, and that are unique to Aboriginal services: the way clients and staff talk to each other; the perception of family; the emphasis on country/mob/where you come from; the value of role-modelling positive behaviour; and the lived experience from Aboriginal Elders or senior staff. OH recognises that healing is not just related to the wellbeing of the individual, but also the wellbeing of the broader community, thus acknowledging the interconnectedness between social, cultural, spiritual and environmental influences of health. These elements are embodied in the red centre of the circle because they are applied across all of the other five core treatment components.

##### Case management

The collaborative process of assessment, planning, facilitation and advocacy to meet an individual’s holistic needs, or *case management,* is an important component to all residential rehabilitation services. In an Aboriginal residential rehabilitation context, case management must also ensure robust partnerships with ACCHOs.

##### Therapeutic activities

The range of *therapeutic activities* implemented at OH comprises individual counselling (predominantly motivational interviewing and cognitive behaviour therapy), in addition to daily psychoeducational groups and weekly 12-Step meetings. Aboriginal-specific therapeutic activities are embedded into program delivery via informal, ad hoc conversations or “yarns” that focus on identity, personal spirituality, an individual’s connection to country, and the value of relationships.

##### Life skills

To ensure clients lead meaningful lives when they return to families and communities, they are encouraged to strengthen a range of *life skills*. Life skills developed or re-established during treatment aims to foster a stronger sense of self through kinships, cultural connection, developing a consistent routine and enhancing personal responsibility from learning work-ready skills.

##### Time out from substances

*Time out from substances* refers to a client’s time away to recuperate from using and/or the interactions with people who encouraged or maintained their substance misuse. Time out from substances therefore aims to provide a client with the time required to focus on improving their physical, mental and spiritual health, largely through developing alternative activities to substance misuse during spare time in preparation for discharge. For instance, being on country or near the river was identified as a key activity that epitomises this core treatment component.

##### Aftercare support

*Aftercare support* aims to provide ongoing support tailored to the client’s needs, allowing for flexibility to “step up” or “step down” to OH or other services, as required. Maintaining a client’s wellbeing after discharge is currently enacted through ongoing relationships with OH staff or linking clients with services and AA groups in their community prior to discharge.

##### Links with services and networks

Links with services and networks is core to OH program delivery as for many clients, as this may be their only point of contact with the health care system. Therefore, links with services to support a client’s physical and mental health needs during treatment is a priority, alongside maintaining parole conditions or supporting clients to undertake withdrawal prior to admission. Broader professional networks across the drug and alcohol residential rehabilitation sector is also important to ensure OH is not isolated from integral knowledge exchange with comparable services, despite its remote geographic location.

##### Staff skills

OH staff must be client-centred, flexible and committed to improving the quality of lives of clients admitted to the service. Therefore, OH strives to employ combination of predominantly local Aboriginal staff with a mix of lived experience and formal qualifications. Staff must also be supported via clinical and cultural support and access to training.

##### Governance, rules and routine

A strong program vision and purpose, as well as a robust, empowered and objective governance structure is required to ensure effective delivery of OH’s service delivery to clients as well as adequate resources. Furthermore, program governance needs to be supported by fair and consistent rules and routine, in addition to ongoing quality improvement and capacity building via collaborative research partnerships.

#### Orana Haven treatment and organisational program logics

A program logic is a depiction of a program designed to clearly align the problem being addressed with what the program will do, and articulate what aspects of the clients and the program will be measured. Two program logics have been developed as a mechanism to operationalisation the core components that summarise OH’s program delivery (Fig. 2). Table 1 relates to the core treatment activities within the OH program and Table 2 relates to the key organisational activities required to maintain effective service provision. Both tables articulate the following:

- *Client or organisational areas of need.* Outlines the primary and secondary client needs that OH aims to target, or the organisational areas of need, as defined in OH’s Strategic Intent;
- *Treatment.* Operationalises and describes associated flexible activities of the central treatment component, five core treatment components, and three organisational components;
- *Mechanisms of change.* Articulates key mechanisms of change for clients/organisation;
- *Process measures*. Specifies key processes to quantify client/organisational change; and
- *Outcomes.* Specifies key outcomes to measure or quantify client or organisational change.

## Discussion

To our knowledge, the process and outcome of researchers working in partnership with a remote Indigenous residential rehabilitation service to define, standardise and operationalise core treatment and organisational components has not been undertaken, or at the very least, has not been extensively published in the peer reviewed literature (James et al., 2017, under review). The Healing Model of Care proposed in this paper articulates that a successful admission to a remote Indigenous drug and alcohol residential rehabilitation service is that as a client’s quality of life and cultural connectedness increases, risky substance use decreases.

### The value of culture

Measuring changes in cultural connectedness and quality of life in conjunction with risky substance use among Indigenous Australians admitted to residential rehabilitation is also consistent with Indigenous peoples’ conceptualisation of health and wellbeing, both in Australia and internationally, which recognises that culture is a key determinant of Indigenous health and wellbeing (NIDAC, 2014; Brady, 1995; Chenhall & Senior, 2013). Strengthening or reconnecting with culture is therefore essential to Indigenous peoples’ healing and recovery from substance misuse as it provides an important protective function (NIDAC, 2014; Chenhall & Senior, 2013; Taylor et al., 2010; McCormick, 2000; Brady, 1995; Torres Stone et al., 2006). This explicit focus on the centrality of culture in treatment is the primary factor that distinguishes Indigenous from non-Indigenous treatment services. It is not to argue that Indigenous people do not benefit from non-Indigenous services, nor that non-Indigenous people do not benefit from Indigenous services, only that outcomes for Indigenous clients in Indigenous services are likely to be optimised by embracing and operationalising the concept of culture in treatment. Having recognised the potential primacy of this concept it now does, of course, require empirical evaluation (James et al., 2017, under review; Chenhall & Senior, 2013; Chenhall & Senior, 2012; Gone & Calf, 2011).

### The value of standardising core components

Defining Indigenous residential rehabilitation programs using standardised core components with flexible activities specific to each service, as articulated in this paper, provides one possible solution to the problem of the inconsistent delivery and diverging views on the appropriateness and efficacy of treatment components. The authors note there are a number of models that could be used to guide the development of services in addition to the logic model framework that the research partnership have utilised in current example, such as Outcomes Star (MacKeith, 2011). However the primary difference of the current research in comparison to other models, is that the research partnership have been able to define the service delivery in concrete terms in a way that is both standardised (core components) and flexible (specific activities). As such, a key strength of this approach is that the definition does not require programs to adhere to a prescribed approach, but provides a structure within which different Indigenous drug and alcohol residential rehabilitation services can categorise preferred treatment activities to their service. For instance, services located in remote areas will have different activities to services in metropolitan or coastal settings. Furthermore, programs in other communities may have more than these core components, but are defined as being comparable to OH if they have these same core components, irrespective of the specific activities developed and delivered to suit the unique circumstances in which they are being implemented.

### The value of standardising outcome measures

Given the reported inconsistency in outcomes measures utilised across Indigenous drug and alcohol residential rehabilitation services both in Australia and internationally (James et al. 2017, under review), the adoption of the program logic framework delineated in this paper may help standardise the outcome measures used in different services. The potential suite of outcome measures would likely increase over time to include other domains such as homelessness, specific health issues, family restoration and community-level benefits of programs (NADA, 2009). Where possible, outcome measures validated for use with Indigenous peoples were selected for the current Healing Model of Care. These included the Growth and Empowerment Measure (GEM; Haswell et al., 2010), the Alcohol Use Disorders Identification Test (AUDIT; Calabria et al., 2014), the Indigenous Risk Impact Screen (IRIS; Schlesinger et al., 2007), the Risk Behaviour Diagnosis Scale (RBD; Gould et al., 2014), and the 10-item Kessler Psychological Distress Scale (K10; Bougie, Arim, Kohen & Findlay, 2016). We recognise other outcome measures, namely the World Health Organization Quality of Life – BREF (abbreviated version; WHOQoL-BREF) is not currently validated for use with Indigenous peoples, but given that health education and behaviour studies are tested for validity and reliability inconsistently (Berry et al., 2013) and there have been no measures designed and validated for use within Indigenous drug and alcohol residential rehabilitation settings, the authors consider this a pivotal area for future research (Stephens et al., 2013; James et al., 2017, under review).

### The value of the CBPR approach

The CBPR approach adopted in this study was found to create a dynamic community-researcher partnership that facilitated meaningful data collection and interpretation over the duration of the 3-year study period. Partnerships between researchers, community members, clients and services, such as the example presented in this paper, therefore have great potential to improve methodological quality and community participation when research skills and community knowledge are integrated to co-design, implement and evaluate community development projects (Munro et al., 2017, under review; Taylor et al., 2010; NIDAC, 2014; Snijder et al., 2015).

### Implications

First, the Healing Model of Care articulated in this paper could be easily be scaled up and applied across other Indigenous drug and alcohol residential rehabilitation services using a similar CBPR framework. By adopting a more standardised approach, the logic model specifically aligns each treatment component and outcome with the mechanism of change for the client or organisation, which then allows for rigorous evaluation and ongoing quality improvement to ensure improved outcomes. As such, this model has the potential to rapidly develop a larger and more rigorous evidence-base to improve outcomes for clients attending Indigenous residential rehabilitation services, both within Australia and internationally, including for Native American or Maori services. It could therefore be adapted and applied to a range of cultural or ethnic minority communities where there may be key components or flexible activities of effective treatment that are specific to their culture. As such, this provides one possible solution to how to provide better care for the large and growing population of Indigenous people with substance dependence transitioning from custody to community. Second, no evaluations published to date have undertaken an economic analysis to weigh the benefits of the treatment approach against its costs (James et al., 2017, under review). This makes it difficult for governments and other agencies to justify funding programs on the basis of a likely economic return for their investment. Therefore, this paper recommends an economic analysis of Indigenous drug and alcohol residential rehabilitation services to methodologically guide future efficiency and resource equity considerations for services, researchers and funding bodies.

### Conclusion

There is a clear lack of rigorous evidence in the Indigenous drug and alcohol residential rehabilitation field due to a number of factors. The description of the CBPR process and the Healing Model of Care presented in this paper provides a possible solution to this problem by defining programs using standardised core components with flexible activities specific to each service. CBPR was found to be integral to enable this research process and has the potential to expand the reach of research across other Indigenous drug and alcohol residential rehabilitation programs. By adopting a more standardised approach, Indigenous drug and alcohol residential rehabilitation services would rapidly develop a larger and more rigorous evidence-base that would likely improve the effectiveness of care provided to all clients accessing these services both in Australia and internationally, but particularly the growing population of Indigenous people with substance dependence transitioning from custody to community.

## Abbreviations

ACCHO: Aboriginal Community Controlled Health Organisation
AH&MRC: Aboriginal Health and Medical Research Committee
AUDIT: Alcohol Use Disorders Identification Test
CO: Carbon monoxide
CQI: Continuous Quality Improvement
GEM: Growth and Empowerment Measure
IPA: Interpretative Phenomenological Analysis
IRIS: Indigenous Risk Impact Screen
K10: 10-item Kessler Psychological Distress Scale
NADA: Network of Alcohol and other Drugs Agency
NDARC: National Drug and Alcohol Research Centre
NH&MRC: National Health and Medical Research
NIDAC: National Indigenous Drug and Alcohol Council
NSQHC: National Safety and Quality in Health Care
OH: Orana Haven Aboriginal Residential Rehabilitation Service
OSR: Aboriginal and Torres Strait Islander Online Services Report
RBD Scale: Risk Behaviour Diagnosis Scale
WHOQoL-BREF: World Health Organization Quality of Life – BREF (abbreviated version)

## References

[CR1] Australian Bureau of Statistics (ABS) (2014). Estimates and projections, Aboriginal and Torres Strait islander Australians, 2001–2026. ABS cat. No. 3238.0. Canberra: ABS.

[CR2] Australian Council of Social Service (ACOSS) (2016). Poverty in Australia 2016.

[CR3] Australian Institute of Aboriginal and Torres Strait Islander Studies (AITSIS) (2012). Guidelines for ethical research in Indigenous studies. AITSIS: Canberra.

[CR4] Australian Institute of Health and Welfare (AIHW). (2011). Substance use among Aboriginal and Torres Strait Islander peoples. Report No.: Cat. no. IHW 40. Canberra; AIHW.

[CR5] Australian Institute of Health and Welfare (AIHW). (2016). Australian Burden of Disease Study: Impact and causes of illness and death in Aboriginal and Torres Strait Islander people 2011. Australian Burden of Disease Study series no. 6. Cat. no. BOD 7. Canberra: AIHW.

[CR6] Australian Institute of Health and Welfare (AIHW). (2017). Aboriginal and Torres Strait islander health organisations: Online services report—Key results 2015–16. Aboriginal and Torres Strait islander health services report no. 8. Cat. No. IHW 180. Canberra: AIHW.

[CR7] Barbour RS (2001). Checklists for improving rigour in qualitative research: A case of the tail wagging the dog?. British Medical Journal.

[CR8] Barry, AE, Chaney, B, Pazza-Gardner, AK, Chavarria, EA. (2013). Validity and reliability reporting practices in the field of health education and behavior: A review of seven journals. *Health Education & Behavior*, *41*, 12–18. 10.1177/1090198113483139 23553350

[CR9] Bessarab, D, & Ng’andu, B. (2010). Yarning about yarning as a legitimate method in Indigenous research. *International Journal of Critical Indigenous Studies*, *3*, 37–50.

[CR10] Brady, M. (1995). Culture in treatment, culture as treatment. A critical appraisal of developments in addictions programs for Indigenous north Americans and Australians. *Social Science and Medicine*, *41*(11), 1487–1498. 10.1016/0277-9536(95)00055-c 8607039

[CR11] Brunette M, Mueser K, Drake R (2004). A review of research on residential programs for people with severe mental illness and co-occurring substance use disorders. Drug and Alcohol Review.

[CR12] Calabria, B, Clifford, A, Shakeshaft, AP, Conigrave, KM, Simpson, L, Bliss, D, Allan, J. (2014). Identifying Aboriginal-specific AUDIT-C and AUDIT-3 cutoff scores for at-risk, high-risk, and likely dependent drinkers using measures of agreement with the 10-item alcohol use disorders identification test. *Addiction Science & Clinical Practice*, *17*(9). 10.1186/1940-0640-9-17. 10.1186/1940-0640-9-17 PMC4158391 25179547

[CR13] Chenhall, R, & Senior, K. (2012). Treating Indigenous Australians with alcohol/drug problems: Assessing quality of life. *Alcoholism Treatment Quarterly*, *30*(2), 130–145.

[CR14] Chenhall, RD, & Senior, K. (2013). “The concepts are universal, it is the picture you paint that is different”: Key issues for Indigenous Australian alcohol and drug residential treatment centres. *Therapeutic Communities: The International Journal of Therapeutic Communities*, *34*(2/3), 83–95.

[CR15] Clifford, A, & Shakeshaft, A. (2017). A bibliometric review of drug and alcohol research focused on Indigenous peoples of Australia, New Zealand. *Canada and the United States. Drug and Alcohol Review*. doi: 10.1111/dar.12510. 10.1111/dar.12510 28334457

[CR16] Cochran, P, Marshall, CA, Garcia-Downing, C, Kendall, E, Cook, D, McCubbin, L, Gover, RM. (2008). Indigenous ways of knowing: Implications for participatory research and community. *American Journal of Public Health*, *98*(1), 22–27. 10.2105/AJPH.2006.093641 PMC2156045 18048800

[CR17] Darke S, Campbell G, Popple G (2012). Retention, early dropout and treatment completion among therapeutic community admissions. Drug and Alcohol Review.

[CR18] Department of Health and Ageing (DOHA) (2013). National Aboriginal and Torres Strait islander suicide prevention strategy.

[CR19] Doyle, M, Butler, T, Shakeshaft, A, Guthrie, J, Reekie, J, Schofield, P. (2015). Alcohol and other drug use among Aboriginal and Torres Strait islander and non-Aboriginal and Torres Strait islander men entering prison in new South Wales. *Health & Justice*, *3*(1), 1–10.

[CR20] Farabee D, Shen H (2004). Antipsychotic medication adherence, cocaine use, and recidivism among a parolee sample. Behavioral Sciences and the Law.

[CR21] Gone JP, Calf Looking PE (2011). American Indian culture as substance abuse treatment: Pursuing evidence for a local intervention. Journal of Psychoactive Drugs.

[CR22] Gould, GS, Watt, K, McEwen, A, Cadet-James, Y, Cough, AR. (2014). Validation of risk assessment scales and predictors of intentions to quit smoking in Australian Aboriginal and Torres Strait islander peoples: A cross-sectional survey protocol. *BMJ Open*, *4*, e004887. doi: 10.1136/bmjopen-2014-004887. 10.1136/bmjopen-2014-004887 PMC4054635 24902729

[CR23] Haswell, MR, Kavanagh, D, Tsey, K, Reilly, L, Cadet-James, Y, Laliberte, A, Wilson, A, Doran, C. (2010). Psychometric validation of the growth and empowerment measure (GEM) applied with Indigenous Australians. *Australian and New Zealand Journal of Psychiatry*, *44*, 791–799. 10.3109/00048674.2010.482919 20815665

[CR24] Heffernan, E, Davidson, F, Andersen, K, Kinner, S. (2016). Substance use disorders among Aboriginal and Torres Strait islander people in custody: A public health opportunity. *Health & Justice*, *4*(12). DOI: 10.1186/s40352-016-0044-8.

[CR25] Honorato, B, Caltabiano, N, Clough, AR. (2016). From trauma to incarceration: Exploring the trajectory in a qualitative study in male prison inmates from north Queensland, Australia. *Health & Justice*, *4*(3). DOI: 10.1186/s40352-016-0034-x. 10.1186/s40352-016-0034-x PMC4819805 27077018

[CR26] Indig D, McEntyre E, Page J (2010). NSW inmate health survey: Aboriginal health report.

[CR27] James, D, Shakeshaft, A, Courtney, R, Munro, A. (2017). A systematic review of Indigenous drug and alcohol residential rehabilitation services: Moving from description to establishing their effectiveness. *Drug and Alcohol Dependence, under review.* 10.2174/1874473711666180404123904 29714152

[CR28] Kinner S, Preen DB, Kariminia A, Butler T, Andrews JY, Stoove M, Law M (2011). Counting the cost: Estimating the number of deaths among recently released prisoners in Australia. Medical Journal of Australia.

[CR29] Kinner S, Wang E (2014). The case for improving the health of ex-prisoners. American Journal of Public Health.

[CR30] Kowanko, I, de Crespigny, C, Murray, H, Ah Kit, J, Prideaux, C, Miller, H, Mills, D, Emden, C. (2009). Improving coordination of care for Aboriginal people with mental health, alcohol and drug use problems: Progress report on an ongoing collaborative action research project. *Australian Journal of Primary Health*, *15*(4), 341–347.

[CR31] Lazarus, L, Shaw, A, LeBlanc, S, Martin, A, Marshall, Z, Weersink, K, Lin, D, Mandryk, K, Tyndall, MW, the PROUD Community Advisory Committee. (2014). Establishing a community-based participatory research partnership among people who use drugs in Ottawa: The PROUD cohort study. *Harm Reduction Journal*, *11*(26). DOI: 10.1186/1477-7517-11-26. 10.1186/1477-7517-11-26 PMC4203893 25307356

[CR32] Leal D, Galanter M, Dermatis H, Westreich L (1998). Correlates of protracted homelessness in a sample of dually diagnosed psychiatric patients. Journal of Substance Abuse Treatment.

[CR33] MacKeith J (2011). The development of the outcomes star: A participatory approach to assessment and outcome measurement. Housing, Care and Support.

[CR34] Marmot, M. (2011). Social determinants of the health of indigenous Australians. *Aboriginal and Islander Health Worker Journal*, *35*(3), 21–22.

[CR35] McCormick RM (2000). Aboriginal traditions in the treatment of substance abuse. Canadian Journal of Counselling.

[CR36] Mooney-Somers, J, & Maher, L. (2008). The Indigenous resiliency project: A worked example of community-based participatory research. *NSW Public Health Bulletin*, *20*(7–8), 112–118. 10.1071/NB09007 19735622

[CR37] Mortlock KS, Dean FP, Crowe TP (2011). Screening for mental disorder comorbidity in Australian alcohol and other drug residential treatment settings. Journal of Substance Abuse Treatment.

[CR38] Munro A, Allan J, Breen C, Shakeshaft A. (2017). “I just feel comfortable out here, there’s something about the place.” Staff and client perceptions of a remote Australian Aboriginal drug and alcohol rehabilitation service. Substance Abuse Treatment, Prevention, and Policy, under review. 10.1186/s13011-017-0135-0 PMC5718008 29208008

[CR39] Munro A, Shakeshaft A, Breen C, Clare P, Allan J, Henderson N. (2017). Understanding remote Aboriginal drug and alcohol residential rehabilitation clients: Who attends, who leaves and who stays? Drug and alcohol review, under review. 10.1111/dar.12656 PMC5969080 29349855

[CR40] National Drug Strategy (NDS) (2015). Intergovernmental committee on drugs National Aboriginal and Torres Strait islander people’ drug strategy 2014–2019.

[CR41] National Health & Medical Research Council (NH&MRC) (2003). *Values and ethics: Guidelines for ethical conduct in Aboriginal and Torres Strait islander Health Research*. Commonwealth of Australia: Canberra.

[CR42] National Indigenous Drug and Alcohol Committee. (2014). Alcohol and other drug treatment for Aboriginal and Torres Strait islander peoples. *Canberra: Australian National Council on Drugs (ANCD)*, 1–23.

[CR43] Network of Alcohol and Other Drug Agencies (NADA). 2009. A Review of Screening, Assessment and Outcome Measures for Drug and Alcohol Settings. Accessed on 10 July 2017 < http://www.drugsandalcohol.ie/18266/1/NADA_A_Review_of_Screening%2C_Assessment_and_Outcome_Measures_for_Drug_and_Alcohol_Settings.pdf>

[CR44] Productivity Commission (2016). *Overcoming Indigenous disadvantage: Key indicators 2016*. Canberra: ACT.

[CR45] Pyett P (2002). Working together to reduce health inequalities: Reflections on a collaborative participatory approach to health research. Australian and New Zealand Journal of Public Health..

[CR46] Queensland Studies Authority (QSA) (2008). *Relationships to country: Aboriginal people and Torres Strait islander people, Indigenous perspectives, RES005*. Queensland Government, Brisbane: QLD.

[CR47] Schlesinger, CM, Ober, C, McCarthy, MM, Watson, JD, Seinen, A. (2007). The development and validation of the Indigenous risk impact screen (IRIS): A 13-item screening instrument for alcohol and drug and mental health risk. *Drug and Alcohol Review*, *26*(2), 109–117. 10.1080/09595230601146611 17364845

[CR48] Shakeshaft A, Petrie D, Doran C, Breen C, Sanson-Fisher R (2012). An empirical approach to selecting community-based alcohol interventions: Combining research evidence, community views and professional opinion. BMC Public Health.

[CR49] Snijder, M, Shakeshaft, A, Wagemakers, A, Stephens, A, Calabria, B. (2015). A systematic review of studies evaluating Australian Indigenous community development projects: The extent of community participation, their methodological quality and their outcomes. *BMC Public Health*, *1154*(15). DOI: 10.1186/s12889-015-2514-7. 10.1186/s12889-015-2514-7 PMC4655078 26590869

[CR50] Stephens, A, Bohanna, I, Graham, D, Clough, AR. (2013). Screening and assessment instruments for use in Indigenous-specific alcohol and drug treatment rehabilitation. *Journal of Tropical Psychology*, *3*, 1–11.

[CR51] Sung H-E, Belenko S, Feng L (2001). Treatment compliance in the trajectory of treatment progress among offenders. Journal of Substance Abuse Treatment.

[CR52] Taylor, K, Thompson, S, Davis, R. (2010). Delivering culturally appropriate residential rehabilitation for urban Indigenous Australians: A review of the challenges and opportunities. *Australian and New Zealand Journal of Public Health*, *34*(Suppl 1), S36–S40. 10.1111/j.1753-6405.2010.00551.x 20618291

[CR53] Torres Stone RA, Whitbeck LA, Chen X, Johnson K, Olson DM (2006). Traditional practices, traditional spirituality, and alcohol cessation among American Indians. Journal of Studies on Alcohol.

[CR54] Wallerstein N, Duran B (2006). Using community-based participatory research to address health disparities. Health Promotion Practice.

[CR55] Weatherburn, D. (2008). The role of drug and alcohol policy in reducing Indigenous over-representation in prison. *Drug and Alcohol Review*, *27*(1), 91–94. 10.1080/09595230701710811 18034386

[CR56] Weatherburn D. (2014). Arresting incarceration: Pathways out of Indigenous imprisonment. Aboriginal Studies Press, Canberra: ACT

[CR57] Wilson I, Graham K, Taft A (2017). Living the cycle of drinking and violence: A qualitative study of women’s experience of alcohol-related intimate partner violence. Drug and Alcohol Review.

[CR58] Windsor LC (2013). Using concept mapping in community-based participatory research: A mixed methods approach. Journal of Mixed Methods Research.

[CR59] Wynne-Jones M, Hillin A, Byers D, Stanley D, Edwige V, Brideson T (2016). Aboriginal grief and loss: A review of the literature. Australian Indigenous Health Bulletin.

