# Correction to: The development of a healing model of care for an Indigenous drug and alcohol residential rehabilitation service: a community-based participatory research approach

**DOI:** 10.1186/s40352-018-0061-x

**Published:** 2018-03-14

**Authors:** Alice Munro, Anthony Shakeshaft, Anton Clifford

**Affiliations:** 1 National Drug and Alcohol Research Centre, University of New South Wales, Sydney, NSW 2052 Australia; 2 University of Queensland, Brisbane, QLD 4072 Australia

## Correction

Upon publication of the original article (Munro et al., 2017), the authors noticed the following errors:

1. A few words are missing in the conclusion of the Abstract. It needs to read:

**Conclusion**: The description of the CBPR process and the Healing Model of Care provides one possible solution about how to provide better care for the large and growing population of Indigenous people with substance misuse issues

2. On page 4, at the top of the page: “Step 1: Effective engagement…” should be “Step 1: Initial engagement...”
3. On page 5, in the middle of first column, the start of the sentence “The semi-structured interviews used ‘yarning’ approach” should be: “The semi-structured interviews used a research ‘yarning’ approach”
4. On page 6, in the section with the sub-heading “Healing through culture and country”, “red centre of circle” should be: “in the centre circle”
5. Table

- The “Aftercare” core treatment component at the bottom of column b is missing
- “b. Intervention” should instead read “b. Treatment”
  Please see the corrected Table 1 below.

6. The second sentence of the Discussion should be: “The Healing Model of Care proposed in this paper is based on the premise that successful treatment in a remote Indigenous drug and alcohol residential rehabilitation service will improve clients’ quality of life and cultural connectedness which will, in turn, be strongly associated with sustained reductions in their risky substance use.”
7. On page 10, the following sentence requires two corrections: “We recognise other outcome measures, namely the World Health Organization Quality of Life – BREF (abbreviated version; WHOQoL-BREF) is not currently validated for use with Indigenous peoples, but given that health education and behaviour studies are tested for validity and reliability inconsistently (Berry et al., 2013) and there have been no measures designed and validated for use within Indigenous drug and alcohol residential rehabilitation settings, the authors consider this a pivotal area for future research (Stephens et al., 2013; James et al., 2017, under review).”

- the first mention of “is” should be “are”
- The reference “Barry et al., 2013” should be “Berry et al. 2013”

8. In the References section, the word “Islander” in the term “Aboriginal and Torres Strait Islander” also should be capitalised. The following references in the reference list need this change to be made:

- AIHW, 2017;
- DOHA, 2013;
- Doyle et al., 2015
- Doyle et al., This also needs a capital “N” in “NSW”
- Gould et al., 2014;
- Heffernan et al., 2016;
- NH&MRC, 2013;
- NIDAC, 2014;
- QSA, 2008.
- Marmot, 2011. This also needs a capital “I” in “Indigenous”

**Table 1:** Orana Haven treatment program logic

a. Client areas of need	b. Treatment		c. Mechanisms of change	d. Process measures	e. Outcomes*
	Core treatment components	Flexible activities			
*Primary client areas of need:* 1. Risky substance use 2. Poor quality of life 3. Poor cultural connection	Healing through culture and country	- Being on country/spiritualty - Developing kinships - Making artefacts, fishing bush medicine	Reconnecting clients to culture and country via activities and strong relationships	No. of clients engaged in regular cultural activities	*Primary outcomes:* 1. Reduced substance misuse (AUDIT/DUDIT* / IRIS* clean urines) 2. Increased quality of life (WHOQoL-BREF*) 3. Increased connection to culture (GEM*)
	Case management	- Referrals to local health services and visiting specialists - Working with corrections - File notes / assessments - Client transport	Clients engaged in the program via positive therapeutic alliance between staff and clients Referrals to AMS to external health and other social services	No. of clients staying in the program for 3 or more mths No. of Aboriginal Health Checks/other referrals No. of kms of transport	
*Secondary client areas of need:* 4. Co-occurring mental illness 5. Criminal justice involvement 6. Chronic physical health needs 7. Tobacco use 8. Unemployed / limited education	Therapeutic activities	- One-on-one counselling - AA, morning, psychoeducational groups - Informal counselling	Improving client quality of life Increased understanding of substance misuse (e.g. triggers) and personal strategies (e.g. motivations, goals, timeout) for reducing misuse	No. of clients maintaining abstinence 3 months post discharge No. of external counselling sessions provided	*Secondary outcomes:* 4. Reduced psychological distress (IRIS* / K10*) 5. Reduction in recidivism (Pre/post criminal justice data) 6. Improved physical health (Pre/post Aboriginal health check outcomes) 7. Reduction in smoking (RBD Scale* / self-report* / CO levels*) 8. Improvement in employment and education (3mth follow-up data)
	Life skills	- Develop daily routine - Positive role-modelling - Redevelop personal responsibility - Vocational courses - Literacy / communication skills	Reconnecting clients to culture and country Relearning daily routine and structure to maintain a healthy lifestyle after discharge Learning and developing work-ready and communication skills	No. of vocational-related courses completed No. of clients achieving individualised life skills goals	
	Time out from substances	- Improve physical wellbeing (eg. sleep routine / nutrition) - Improve mental / spiritual wellbeing - Smoking cessation	Identify and engage in positive alternative activities to substance use to learn how to take time out from substance substances	No. of clients engaging in regular exercise / cultural activities No. of clients quitting or reducing smoking	
	Aftercare support	- Referrals to services post-discharge (eg. ACCHOs) - Provide a list of support services in client’s community (eg. AA) - Ongoing phone contact	Continue to access treatment and care required to maintain improved health and wellbeing post discharge Developing aftercare program post discharge from treatment	No. of clients maintaining abstinence/not involved in crime post discharge No. of clients participating in aftercare (eg. phone calls, assessments, visits)	

*Measured at admission, mid, discharge and 3mths post discharge from the OH program
